# Dexmedetomidine–Ketamine or Dexmedetomidine–Midazolam Nebulised Drug Combination as a Premedicant in Children: A Randomised Clinical Trial

**DOI:** 10.5152/TJAR.2022.21298

**Published:** 2022-10-01

**Authors:** Tanvi Dhiman, Versha Verma, Ravinder Kumar Verma, Shelly Rana, Jai Singh, Isha Badhan

**Affiliations:** 1 Department of Anaesthesia, Dr. Rajendra Prasad Government Medical College Kangra, Himachal Pradesh, India

**Keywords:** Dexmedetomidine, ketamine, midazolam, premedication

## Abstract

**Objective:**

This study was designed to evaluate the clinical efficacy of 2 low-dose nebulised drug combinations of dexmedetomidine–ketamine and dexmedetomidine–midazolam as a premedication in children scheduled for surgery under general anaesthesia.

**Methods:**

Sixty children classified as American Society of Anesthesiologists physical status I, aged between 3 and 10, listed to undergo elective surgeries under general anaesthesia were enrolled in this prospective, randomised, and double-blind trial. Patients were randomly allocated to receive nebulised premedication approximately 30 minutes before the induction of anaesthesia. Group DK (n = 30) received combined nebulised dexmedetomidine and ketamine (1 μg kg^−1^ + 1 mg kg^−1^) and the dexmedetomidine-midazolam (DM) group (n = 30) received combined nebulised dexmedetomidine and midazolam (1 μg kg^−1^ + 0.1 mg kg^−1^). All children were anaesthetised with a protocolised anaesthesia technique. The primary end point was the level of sedation when the child was first seen in the operating room 30 minutes after nebulisation. The secondary end points were parental separation and ease of induction, ease of acceptance of IV cannula, mask acceptance, postoperative analgesia, and wake-up behaviour.

**Results:**

Studied groups were comparable in demographic data (age, weight, and sex) and duration of anaesthesia. Level of sedation at 30 minutes was significantly greater in the DM group than in the DK group (*P*  = .013) while the two were comparable in parental separation and ease of induction (*P*  = .808). Group DK exhibited superior ease of acceptance of IV cannula (*P*  = .001), mask acceptance score (*P*  = .001), and postoperative analgesia (*P*  = .021). Hemodynamic parameters and oxygen saturation remained comparable at all time intervals as also the wake-up behaviour.

**Conclusions:**

The nebulised combination of low-dose ketamine and dexmedetomidine was a superior combination producing acceptable sedation with enhanced ease of IV acceptance, mask acceptance, and postoperative analgesia in children.

**Main Points**

- Children undergoing procedures require preoperative sedative medication.
- Diverse routes of administration are available, each with their own merits and demerits.
- Children who received nebulisation with dexmedetomidine–ketamine combination offered acceptable sedation with superior ease of IV acceptance, mask acceptance, and postoperative analgesia and hence a better combination.

## Introduction

Premedication is an indispensable aspect of paediatric anaesthesia which, when ideal, allows easy separation of the child from the parent for induction and facilitates a smooth conduct of anaesthesia.^1^ Stranger anxiety, fear of parental separation, and alien operating room environment all contribute to psychological trauma before surgery in 70% of children.^2^ Emergence agitation (EA) is proportionate to the level of preoperative anxiety preceding surgery.^3^ Postoperative pain associated with preoperative anxiety may hinder the recovery of the patient.^4^

Premedication administered via oral routes has a delayed onset while intramuscular, rectal, and intravenous routes are painful and frightening for children. Nebulisation route offers a relatively simple, non-invasive, and convenient method which also diminishes the first-pass metabolism of the drug with minimal absorption into the systemic circulation.

Dexmedetomidine is a potent, highly selective α2 agonist that produces sedation and analgesia by its action on locus ceruleus via inhibiting the enzyme adenylate cyclase. It has a shorter half-life and produces a sleep-like state.^5^ The potential advantage of this drug is that there is no respiratory depression or confusional state after the procedure making it popular in paediatric patients for premedication and procedural sedation. There exists a possibility to give dexmedetomidine in combination with other drugs to circumvent unfavourable effects of dexmedetomidine like bradycardia and hypotension, which usually occur when used in higher doses alone.

Ketamine is an N-methyl-D-aspartate receptor antagonist. The primary action is due to the central dissociation of the cortex from the limbic system. It provides sedation and analgesia and also preserves upper airway muscular tone and respiratory drive.^6^ Ketamine has been widely used for sedation and analgesia in children in several non-operative offsite situations, like in emergency ward, radiation, radiology suites, etc.^7^

The possible unwanted side effects of ketamine include excessive salivation, restlessness, anxiety, and postoperative vomiting.^8^

Midazolam is another frequently used premedication in children. It is water soluble but becomes lipid soluble at physiological pH levels, allowing it to cross the nasal mucosa with a quick onset of action. Midazolam is not only advantageous in reducing preoperative anxiety but it also improves recovery from anaesthesia attributable to its anxiolysis and anterograde amnesia.

The combination of midazolam with dexmedetomidine made sense as midazolam lacks the analgesic property which is provided by dexmedetomidine and hence the individual disadvantage of each drug can be counterbalanced when used in combination. The reduction in the required dose further minimises the adverse effects.

Dexmedetomidine primarily acting in the locus coeruleus of the central nervous system induces electroencephalogram activity mimicking natural sleep while midazolam is a GABAergic sedative drug.^9^ Hence, the combination of these 2 agents could enhance the sedative efficacy. The combination of dexmedetomidine with ketamine is pharmacodynamically wise, as they have contrasting haemodynamic effects, one increases blood pressure (ketamine) and the other (dexmedetomidine) decreases the same. The faster onset time of ketamine also counterbalances the slow onset time when dexmedetomidine is used alone.^10^

The purpose of this study was to evaluate the clinical efficacy of 2 low-dose nebulised drug combination regimes of dexmedetomidine–ketamine and dexmedetomidine–midazolam as premedication in children undergoing surgical procedures under general anaesthesia.

## Methods

This prospective, randomised, double-blind study was conducted from June 2018 to December 2019 after obtaining approval from the ethics committee of Dr Rajendra Prasad Government Medical College Kangra (IEC:2017/08). The trial was registered prior to enrollment of patients in the clinical trial registry of India (CTRI/2018/05/013772). A written and informed consent was obtained from the parents of the children after explaining the nature of the study. Sixty children in the age group of 3-10 years of either gender, belonging to American Society of Anesthesiologists status I, posted for elective surgical procedures involving upper limb procedures scheduled under general anaesthesia, were included. Children with known allergies to the study drugs, active respiratory infection, cardiac dysfunction, mental retardation, prematurity, nasal deformities like spur, deviated nasal septum, and duration of surgery of more than 2 hours duration were excluded from the study.

During the preoperative visit, the patient’s detailed history, general physical examination, and systemic examination were carried out. Routine investigations like haemoglobin, bleeding time, and clotting time were done in all patients. Nil per oral orders were as per standard protocol.

Randomisation was done by a computer-generated randomised number table. Random numbers were enclosed in a sealed opaque envelope and opened by an independent investigator, not involved in the observation or the administration of the anaesthesia, to know the study drug combination to be administered. Dexmedetomidine at a concentration of 100 µg mL^−1[Bibr b1-tjar-50-5-380]^, ketamine at a concentration of 50 mg mL^−1^^,^ and midazolam at a concentration of 1 mg mL^−1^ were used. The study drugs were prepared without dilution in identical syringes. The volume of dexmedetomidine, ketamine, and midazolam were 0.01 mL kg^−^^1^, 0.02 mL kg^−1[Bibr b1-tjar-50-5-380]^ and 0.1 mL kg^−1^, respectively. The final amount was 0.03 mL kg^−1^ for dexmedetomidine–ketamine and 0.1 mL kg^−1^ for dexmedetomidine–midazolam, which was diluted with 3 mL of 0.9% saline before administration through nebuliser. A standard hospital jet nebuliser, Mehar Ready Neby piston compressor nebuliser, with an appropriate size facemask allowing a continuous flow of 100% oxygen at 6 L min^−1^ for 10-15 minutes (30 minutes before the anticipated time of induction) was used. The face mask was held comfortably by the older children and either of the parents held it for the younger ones. Treatment was stopped when the nebuliser began to sputter. The observer anaesthesiologist, who collected the data, was blinded to the test drug combination administered through the nebulisation.

According to the random number, the patients were allocated to 1 of the 2 groups. Group DK received dexmedetomidine 1 µg kg^−1^ and ketamine 1 mg kg^−1^ in nebulised form and group DM received dexmedetomidine 1 µg kg^−1^ and midazolam 0.1 mg kg^−1^ in nebulised form in the preoperative area. Continuous monitoring of heart rate (HR) and SpO_2_ was done every 5 minutes in the preoperative period by the independent observer.

After 30 minutes of the nebulisation, ease of parent separation of the child was assessed using the parental separation anxiety scale;^11^ a 4-point scale: 1 = easy separation, 2 = whimpers, but is easily reassured, not clinging, 3 = cries and cannot be easily reassured, but not clinging to parents, and 4 = crying and clinging to parents. A score of 1 or 2 was classified as an acceptable separation, whereas scores of 3 or 4 as difficult separations from the parents.

In the operation room (OT), child′s level of sedation was assessed using 5-point scale^11^ (sedation score): 1 = agitated, 2 = alert, 3 = calm, 4 = drowsy, and 5 = asleep. A score of 3 and above was considered acceptable sedation.

A 4-point scoring system devised by Gharde et al^12^ was used for evaluation of acceptance of the IV cannula: poor (terrified, crying), fair (fear of needle, not reassured), good (slight fear of needle, easily reassured), and excellent (unafraid, accepts IV cannula readily). Excellent and good were taken as “satisfactory” IV acceptance.

A peripheral intravenous line was established with appropriate size cannula in non-dominant hand of children with a satisfactory score. In others, an intravenous line was established after inhalation induction. Patients were monitored with standard 3 lead electrocardiography, HR, non-invasive blood pressure, and pulse oximeter.

Child’s ability to accept the anaesthesia mask was also assessed using the mask acceptance scale (MAS; 4-point Likert scale)^11^ 1 = excellent (unafraid, cooperative, accepts mask readily), 2 = good (slight fear of mask, easily reassured), 3 = fair (moderate fear of mask, not calmed with reassurance) acceptance, and 4 = poor (terrified, crying, or combative). Patients who had MAS of 1 or 2 were considered satisfactory acceptance of the anaesthesia mask; scores of 3 or 4 were considered not satisfactory.

Inhalation induction with 30% oxygen in 70% nitrous oxide with sevoflurane in a concentration of 8% was done. Once the intravenous line was secured, injection glycopyrrolate 10 μg kg^−1^ was given. The airway was secured with appropriate size Igel, facilitated within propofol 1 mg kg^−1^ and atracurium 0.5 mg kg−1. Anaesthesia was maintained with oxygen (33%)-nitrous oxide-isoflurane. Analgesia was provided with intravenous fentanyl (1-2 μg kg^−1^) and paracetamol (10-15 mg kg^−1^). At the end of surgery, children were reversed with neostigmine 50 μg kg^−1^ and glycopyrrolate 10 μg kg^−^1 and were transferred to post-anaesthesia recovery room and monitored.

In the postoperative period, EA was assessed using the Watcha scale.^11^ A score of more than 2 indicated the presence of EA.

For analgesia, the assessment was done by the Faces Pain Scale-Revised.^13^ Faces scales require selecting a picture of a face that represents one’s pain intensity. Children were monitored for 1 hour in PACU for pain relief. Rescue analgesia with injection fentanyl 0.5-1 μg kg^−1^ was given to children who had faces scale of 4 or more. The primary end-point of the study was the level of sedation when the child is first seen in the OR. The secondary end points included parental separation and ease of induction, ease of acceptance of IV cannula, mask acceptance, postoperative analgesia, sedation at emergence, and wake-up behaviour.

## Statistical Analysis

Based on a previous study,^14^ a minimum of 22 patients in each group should be sufficient to detect a difference between means of the sedation score of 1, assuming a standard deviation (SD) of 0.5 with a power of 80%, and a 2-sided type I error of 5%. Data were presented as frequency, mean, and SD whenever applicable. Categorical variables between 2 groups were compared using the chi-square test. A*P* value <.05 was considered significant. Statistical analysis was performed using Statistical Package for the Social Sciences version 21.0 (IBM Corp.; Armonk, NY, USA).

## Result

**Figure 2.**
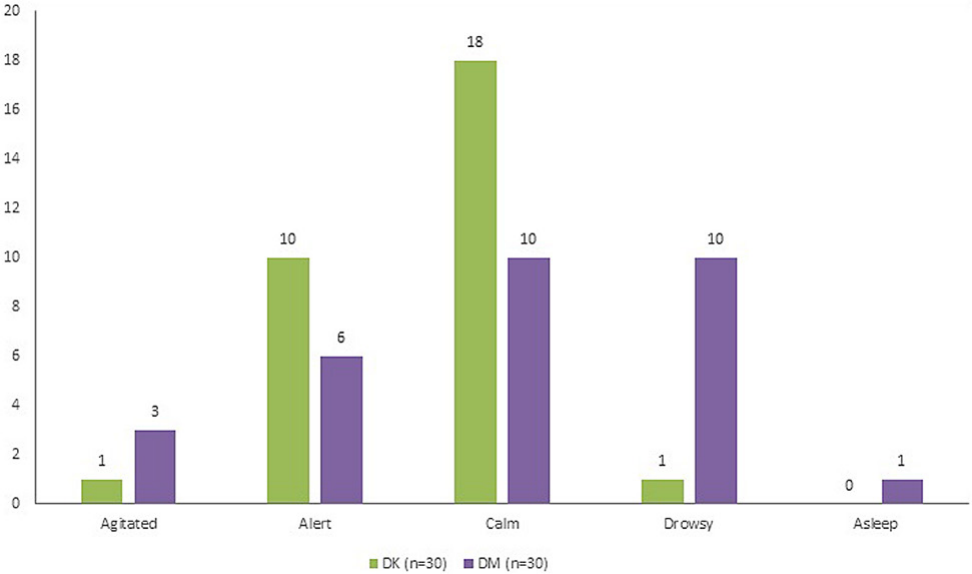
Sedation level scale among 2 groups.

The age, weight, and duration of surgery were comparable between the groups. There was male predominance in both the groups (Table 1). After nebulisation, 63.3% patients in the DK group and 70% patients in the DM group were adequately sedated (a score of 3 and above). The sedation score was significantly more for the DM group compared to the DK group (*P *= .013) (Figure 2).

**Figure 3.**
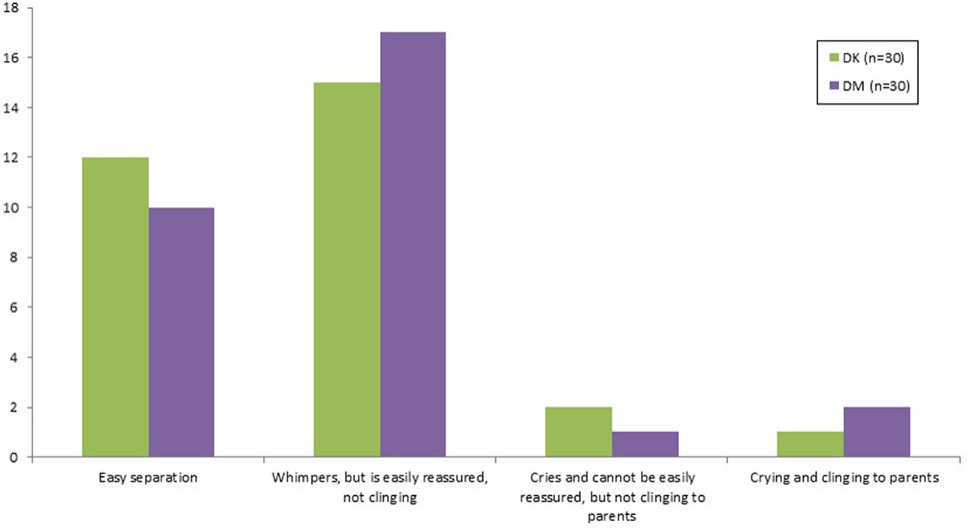
Parental separation and ease of induction scale.

In present study, both groups had an acceptable (classified as a score of 1 or 2) parental separation and ease of induction scores with 90% children in the DK group and 89.9% children in the DM group having acceptable scores. This was, however, statistically insignificant (*P *= . 808) (Figure 3).

**Figure 4.**
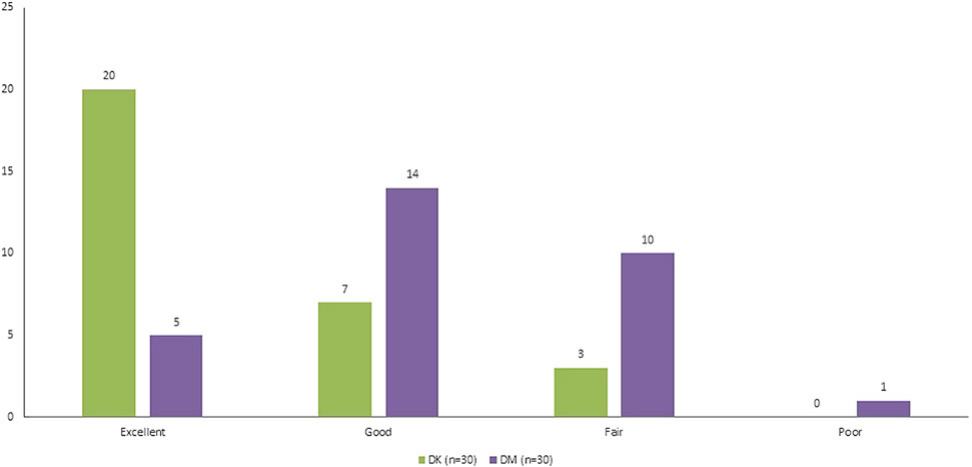
Ease IV acceptance scale among 2 groups.

**Figure 5.**
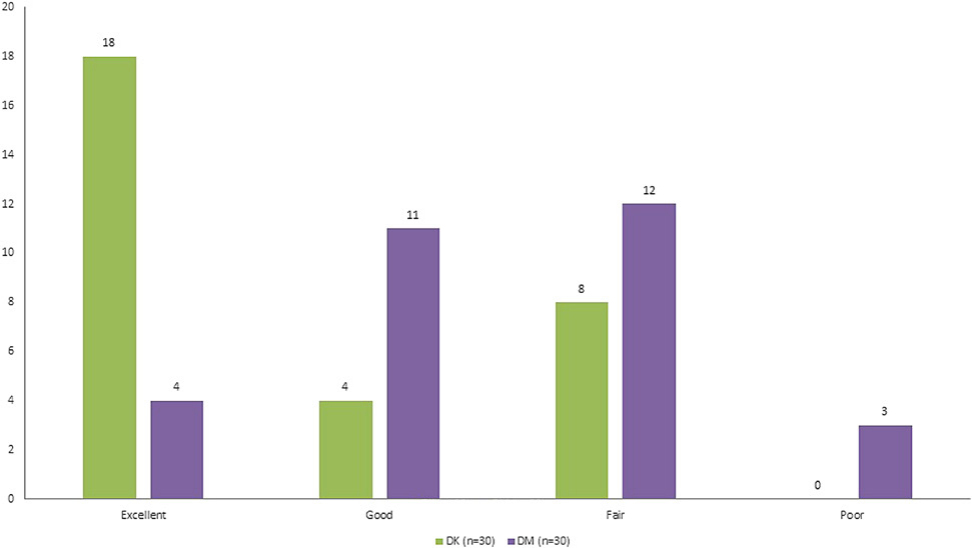
Mask acceptance scale among 2 groups.

**Figure 6.**
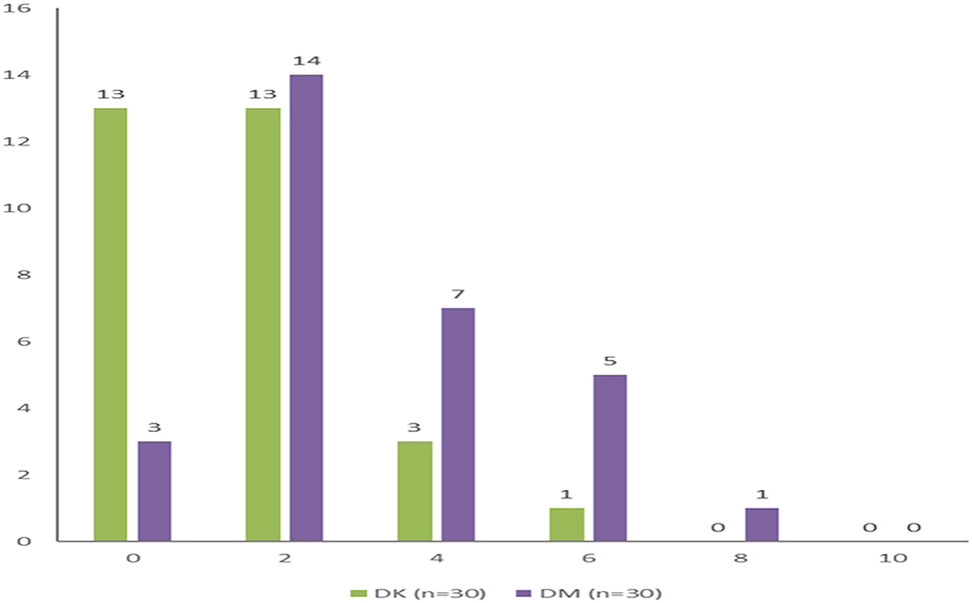
Faces pain scale among both groups.

Ease of intravenous acceptance was observed to be satisfactory in 90% of the patients in the DK group and 64% patients in the DM group. Thus, there was significant difference in intravenous acceptance between both the groups (*P* = .001) (Figure 4). Mask acceptance was satisfactory in 73% of the patients of the DK group and in 50% of patients of the DM group which was statistically significant (*P* = .001) (Figure 5). There was significant difference in pain scores between both groups, that is, DK and DM, with superior analgesia observed in DK group (*P*  = .021) (Figure 6).

**Figure 7.**
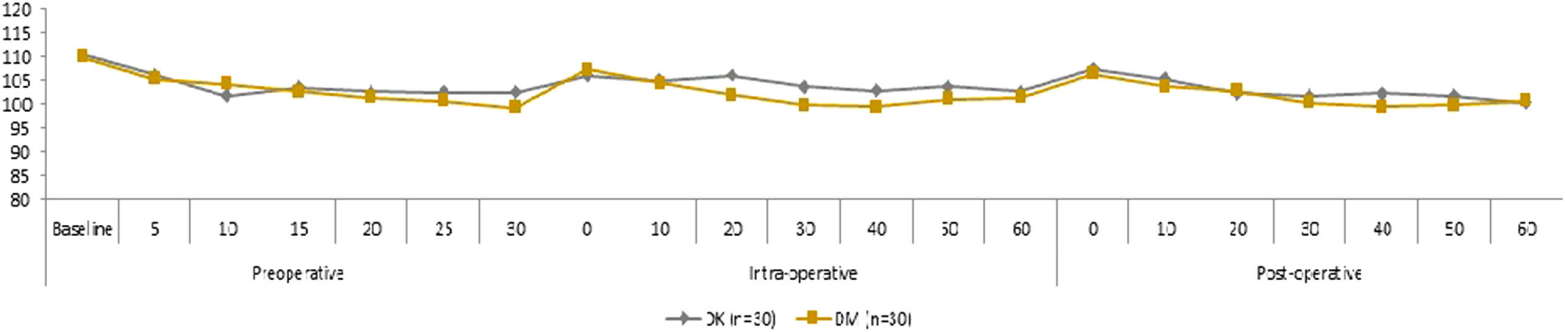
Trends of change in heart rate among 2 groups.

**Figure 8.**
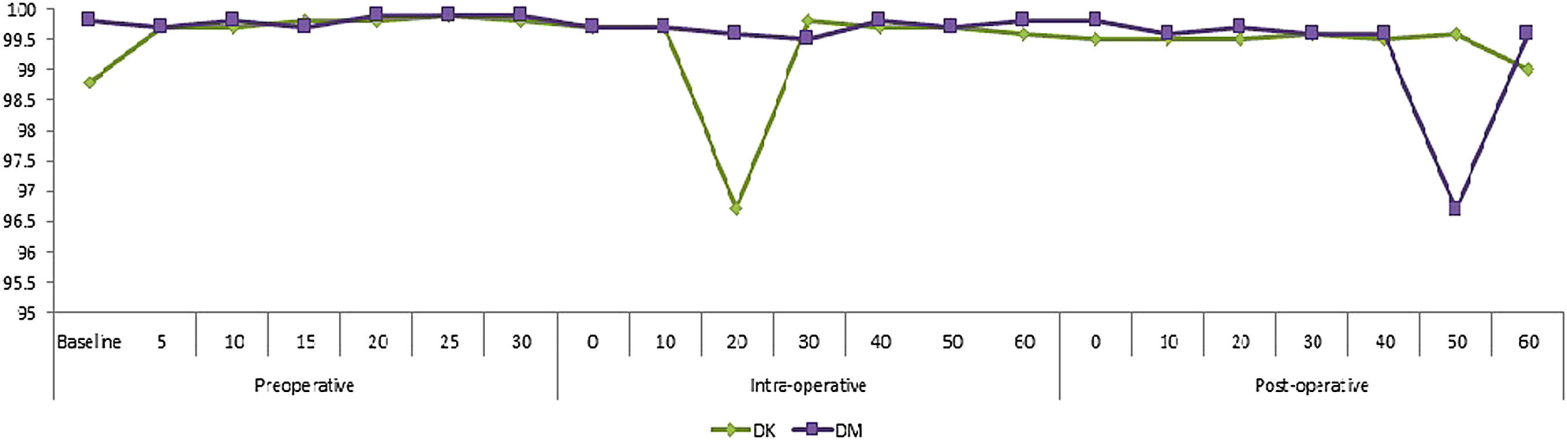
Trends in change of oxygen saturation among 2 groups.

**Figure 9.**
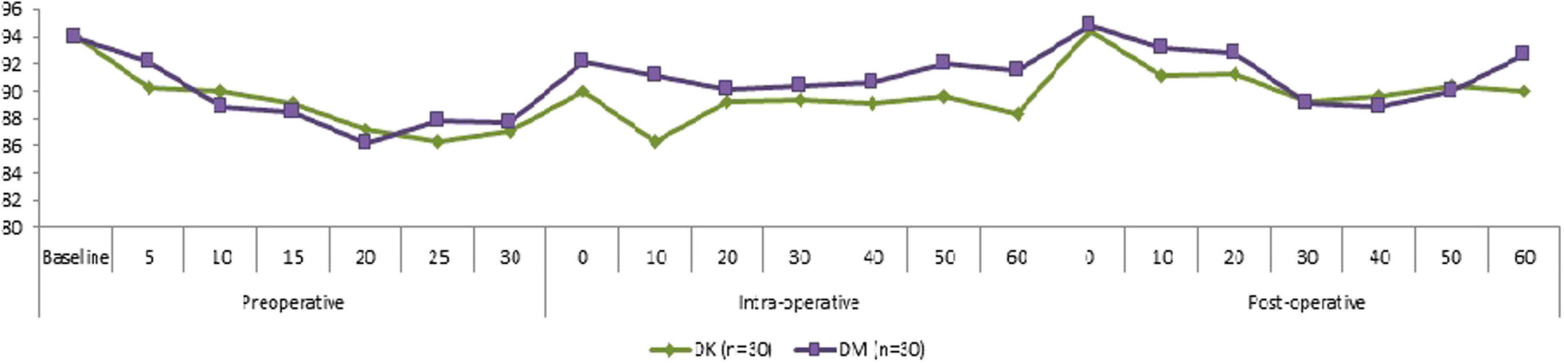
Trends in change of MAP among two groups.

It was observed that SPO_2_, HR, and mean arterial pressure (MAP) was comparable at all the time points before induction and intra-operatively (*P* > .05) (Figure 7-9). In the post-operative period, 20% patients in DK group and 17% patients in DM group were crying post-operatively. Hence the post-operative behaviour score among both the groups was comparable (*P*  = .315).

Minor adverse effects like emergence delirium and nystagmus in the perioperative period were significantly lower in the DM group in comparison to the DK group (*P*  = .001), but these were clinically insignificant.

## Discussion

Premedication in small children often has been a daunting task for the anaesthetist. Nebulisation provides a safe, convenient, atraumatic, and comfortable means of premedication to children compared to other routes especially when an intravenous line has not been secured. Nebulised drug administration has been favoured over intranasal administration, as the intranasal route causes transient nasal irritation, cough, vocal cord irritation, or laryngospasm.

Midazolam, dexmedetomidine, and ketamine all have been used for premedication in children through nebulisation often individually and rarely in combination. Nebulised dexmedetomidine has rapid drug absorption through nasal, respiratory, and buccal mucosa with bioavailability of 65% through the nasal mucosa and 82% through the buccal mucosa.^15^

The hypothesis of the current study was that combining these drugs would provide a better option than using them individually, thereby combating not only the side effects caused by each individual drug by decreasing dose but also improve the efficacy due to synergistic effect. Hence, in the current study, lower doses of each drug were used in combination for example dexmedetomidine 1 µg kg^−1^, ketamine 1 mg kg^−1^, and midazolam 0.1 mg kg^−1^ than the conventionally higher doses when these drugs were used alone.^16^

In the present study, 63.3% patients in the DK group and 70% patients in the DM group were adequately sedated on arrival in OT. The sedation was significantly better with the DM group than the DK group which can be attributed to dexmedetomidine and midazolam acting synergistically. Children in the DK group were however found to be more calm (60%), whereas children in the DM group were both equally calm (33%) and drowsy (33%). This suggests that both the drug combinations provided effective anxiolysis. This finding was in accordance with the study conducted by Abdel-Ghaffar et al^16^ where they compared the efficacy of nebulised dexmedetomidine, ketamine, and midazolam for sedative premedication, a significant difference midazolam>dexmedetomidine>ketamine (*P*  = .000) was seen in the sedation score with midazolam proving to be superior. The sedation produced varied from mild dissociation (ketamine group), as opposed to mild to moderate (dexmedetomidine group) and moderate (midazolam group) sedation.

A similar study by Neville et al^17^ compared anxiolysis with intranasal dexmedetomidine versus intranasal midazolam for paediatric laceration repairs in the emergency department. They also concluded that intranasal dexmedetomidine had a similar performance to intranasal midazolam except that patients who received dexmedetomidine were more calm at the time of positioning for the procedure.

Zanaty and El Metainy^14^ conducted a comparative evaluation of nebulised dexmedetomidine, nebulised ketamine, and their combination as premedication for outpatient paediatric dental surgery. They concluded that a nebulised combination of low-dose ketamine and dexmedetomidine produced more satisfactory sedation and provided a smoother induction of general anaesthesia than nebulised ketamine or dexmedetomidine alone. This finding was similar to the present study.

The parental separation score and ease of induction score between both the groups were comparable in the current study. The combination of DK and DM groups has 90% and 89% of ease in parental separation, respectively; hence both drug combinations fared equally efficacious for parental separation and ease of induction score. This finding was similar to a study conducted by Zanaty and El Metainy.^14^ They also observed that the combination of nebulised dexmedetomidine–ketamine had better parental separation and ease of induction than any single drug, dexmedetomidine or ketamine used alone.

In the present study, mask acceptance was superior with the DK group than the DM group. In the study by Abdel-Ghaffar et al^16^ mask acceptance was significantly better with dexmedetomidine followed by ketamine and midazolam, respectively. Zanaty and El Metainy^14^ also reported superior mask acceptance with nebulised dexmedetomidine and ketamine combination when compared to either drug given alone via the same route.

In the present study, the ease of IV cannulation was significantly higher in the DK group than in the DM group. Correspondingly, in the study conducted by Qiao et al,^18^ the investigators compared oral ketamine and intranasal dexmedetomidine (DK) combination with intranasal dexmedetomidine or oral ketamine. They also concluded that the ease of IV cannulation was better with group DK than group D alone. This is probably owing to the analgesic effect of ketamine.

Postoperative behaviour in the study by Abdel-Ghaffar et al^16^ observed that the incidence of EA was found significantly lower with dexmedetomidine–ketamine than with midazolam or ketamine alone. Similarly, in the current study, this score was comparable among both the groups as 80% of patients in the DK group while 83.33% patients in the DM group were either asleep or calm and the remaining were crying but were consolable at extubation. This was probably due to the effect of dexmedetomidine that reduced EA in the postoperative period.

Postoperative pain scores were lower in the DK group than in the DM in the present study. This was probably due to the analgesic property of ketamine. Similar results were shown by Zanaty and El Metainy^14^ where pain scores were significantly lower with DK than K or D group.

Plambech and Afshari^19^ and HS Abdel-Ghaffar et al^16^ showed that hypotension and bradycardia are the most common adverse events associated with dexmedetomidine and that respiration is only slightly affected. In contrast, in the current study, children in both groups maintained haemodynamics (MAP and HR) and oxygen saturation at all times preinduction, intraopratively, and well into the postoperative period. This could be attributed to a lower dose of dexmedetomidine used in our study.

A potential weakness of the study is that sedation was observed only at arrival in OT rather than at more frequent intervals which could have provided data as regards to the onset of sedation. Second, parental satisfaction scores should have been also incorporated. Also, the results cannot be extrapolated to high-risk children with comorbidities. More randomized controlled trail (RCTs) are required to establish its safety and superiority over other routes for this purpose.

## Conclusion

Premedication via nebulisation with dexmedetomidine–ketamine combination is superior to dexmedetomidine–midazolam as it provided comparable sedation, with enhanced ease of IV acceptance, mask acceptance, and postoperative analgesia in paediatric patients undergoing surgery under general anaesthesia, with minimal side effects. The nebulised route for premedication in children is relatively unexplored and further drug combinations and dose-finding studies are warranted.

## Figures and Tables

**Figure 1.**
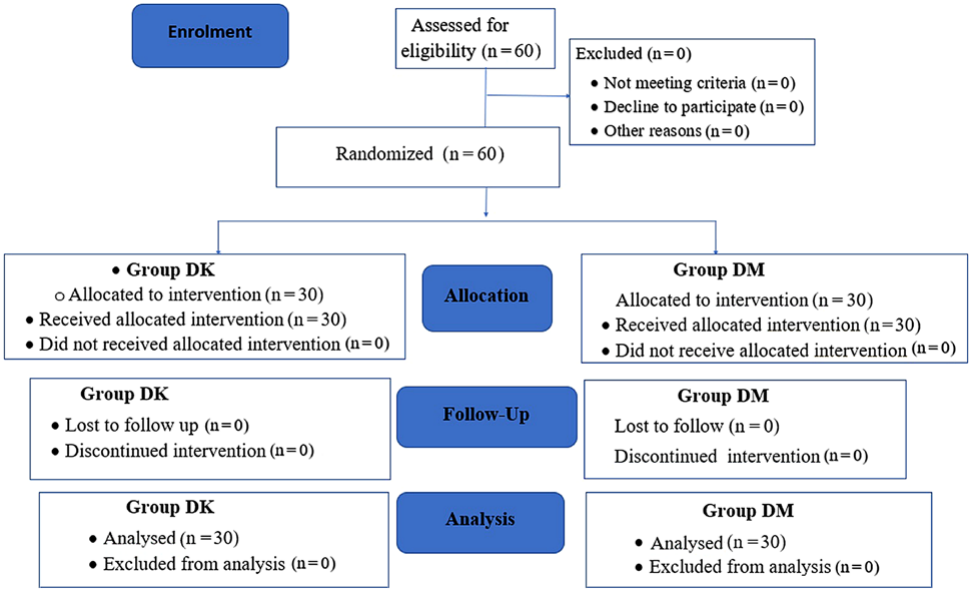
Consolidated standards of reporting trials flow diagram illustrating the patient progress through the study.

**Table 1.** Subject Characteristics and Clinical Data Among 2 Groups

Characteristics	Group DK	Group DM	*P*
Age (years)	6.58 ± 1.78	6.30 ± 1.89	.549
Sex (male : female)	24 : 6	25 : 5	.739^#^
Weight (kg)	16.60 ± 4.43	15.96 ± 4.81	.598
Duration of anaesthesia (mins)	71.33 ± 11.37	70.0 ± 5.87	.570
Sedation score (agitated/alert/calm/drowsy/asleep)	1/10/18/1/0	3/6/10/10/1	.013^#^
Parental separation and ease of induction score (easy separation/whimpers, but is easily reassured, not clinging/cries and cannot be easily reassured, but not clinging to parents/crying and clinging to parents)	12/15/2/1	10/17/1/2	.808^#^
Ease of IV acceptance (excellent/good/fair/poor)	20/7/3/0	5/14/10/1	.001^#^
Mask acceptance (excellent/good/fair/poor)	18/4/8/0	4/11/12/3	.001^#^
Post-op behavior (asleep/calm/crying but can be consoled/crying but cannot be consoled/agitated and thrashing around)	22/2/6/0/0	19/6/5/0/0	.315^#^
Faces pain scale (0/2/4/6/8/10)	13/13/3/1/0/0	3/14/7/5/1/0	.021^#^

Data are expressed as number, mean, and standard deviation; ^#^Chi-square test.
